# Right ventricular dyssynchrony predicts outcome in pulmonary arterial hypertension when assessed in multiple cardiac magnetic resonance views

**DOI:** 10.1016/j.jocmr.2024.101103

**Published:** 2024-09-24

**Authors:** Anthony Lindholm, Barbro Kjellström, Göran Rådegran, Håkan Arheden, Ellen Ostenfeld

**Affiliations:** a Department of Clinical Sciences Lund, Clinical Physiology and Skåne University Hospital, Lund University, Lund, Sweden; b Department of Clinical Sciences Lund, Cardiology, and the Section for Heart Failure and Valvular Disease, Skåne University Hospital, Lund University, Lund, Sweden

**Keywords:** Strain, Magnetic resonance, Prognosis, Pulmonary hypertension, PAH

## Abstract

**Background:**

Right ventricular (RV) dyssynchrony or post systolic contraction (PSC) causes inefficient pumping and has not been investigated as a prognostic marker in pulmonary arterial hypertension (PAH).

The objective was to investigate if RV dyssynchrony and PSC are prognostic markers of transplantation-free survival in PAH and if multiple RV views improve prognostication.

**Methods:**

Patients with PAH undergoing cardiovascular magnetic resonance between 2003 and 2021 were included. For strain analysis, endocardial end-diastolic RV contours were delineated in RV three-chamber (RV3ch), four-chamber (4ch), and midventricular short-axis (SAX) slice. RV dyssynchrony was defined as the standard deviation of time to peak strain in the walls from one (4ch), two (4ch and SAX), or three views (4ch, SAX, and RV3ch). PSC was defined as peak strain occurring after pulmonary valve closure. Outcome was defined as death or lung transplantation.

**Results:**

One hundred and one patients (58 ± 19 years, 66% (67/101) women) were included. Median follow-up was 37 [51] months. There were 60 events (55 deaths and 5 lung transplantations). Outcome was associated with RV dyssynchrony from three views and with RV strain in 4ch. An increase in RV dyssynchrony—in three views—by 1% was associated with a 10% increased risk of lung transplantation or death. There was no association between outcome and RV dyssynchrony in one or two views nor with PSC.

**Conclusion:**

RV dyssynchrony in three views was associated with outcome in PAH, whereas assessing dyssynchrony from one or two views and PSC was not. This implies that assessment of multiple instead of single RV views could potentially be used for prognostication in PAH.

## Graphical abstract

Examples of strain delineations from four-chamber, midventricular short-axis slice, and right ventricular three-chamber view in the upper panel. Strain was measured on six walls from the three views. Strain curves from the six walls, represented by their respective colors, are plotted in the lower left panel. Dyssynchrony from three views (six walls) was associated with lung transplantation or death in Cox regression analysis both in univariate analysis and in bivariate analysis with age, lower right panel

**Figure:**
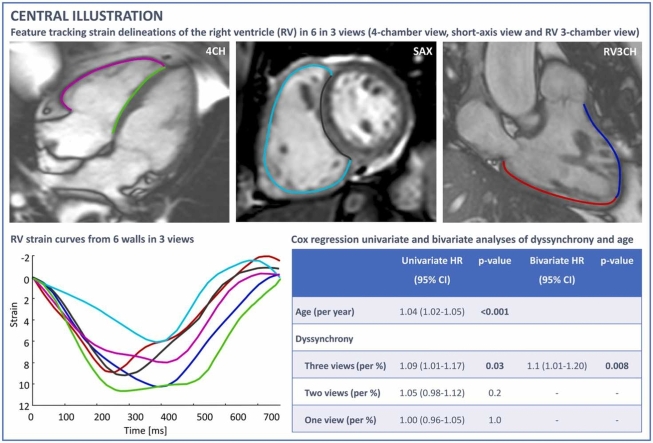

## 1 Introduction

Pulmonary arterial hypertension (PAH) alters right ventricular (RV) function, leading to high morbidity and mortality [1]. Changes in RV function are prognostic of outcome in PAH, and RV function is typically measured by ejection fraction (EF) and global longitudinal strain (GLS) [1], [2], [3], [4], [5]. While EF and GLS reflect global changes, evaluation of inefficient contractions such as intra-ventricular dyssynchrony and post systolic contraction (PSC) are not addressed [6], [7]. Dyssynchrony is regional systolic contractions occurring at different time points of the cardiac cycle [8], [9], and PSC is contraction occurring after pulmonary valve closure [10].

Intra-ventricular dyssynchrony and PSC have mainly been measured in the four-chamber (4ch) view with echocardiography [8], [9]. As the 4ch view includes the interventricular septum, this is prone to left ventricular (LV) interference. The RV three-chamber (RV3ch) view includes only free walls of the RV, and not the septum, why adding RV3ch to dyssynchrony assessment could improve the diagnostics of RV dysfunction. Evaluation from multiple RV views has been suggested to be a more comprehensive measure of RV function [7], [11]. However, the prognostic implications of RV dyssynchrony or PSC have not yet been investigated in PAH and it is not known if RV assessment from multiple views has a prognostic value.

Therefore, the primary aim of this study was to determine if RV dyssynchrony and PSC are prognostic markers for transplantation-free survival in PAH. The secondary aim was to investigate if a comprehensive assessment from multiple views improves the prognostication by using cardiac magnetic resonance (CMR).

## 2 Method

### 2.1 Study population

Patients diagnosed with PAH from 2003 through 2021 were retrospectively included in the study. All patients underwent a CMR on clinical indication at time of diagnosis or at follow-up and were over 16 years old at the time of CMR. RV views for dyssynchrony and PSC assessment were RV3ch, 4ch, and short-axis (SAX) slice. Exclusion criteria were septal defects, atrial fibrillation, significant valvular disease, insufficient cine image quality, or less than two of three RV views available.

Outcome was defined as either lung transplantation or death, and data were acquired from medical journals. The end of follow-up was April 30, 2022.

The study complies with the Declaration of Helsinki and was approved by the Regional Ethical Review Board of Lund. Written informed consent was acquired from all participants before the study procedures.

### 2.2 Right heart catheterization

Right heart catheterization was performed at rest in the supine position. A triple-lumen 7F balloon-tipped Swan-Ganz catheter was inserted in the right internal jugular vein using an 8F sheath introducer with local anesthesia. Pulsatile as well as mean right atrial pressures, pulmonary arterial pressures, and pulmonary artery wedge pressures were acquired during free breathing over several heartbeats. Cardiac output was measured by thermodilution. Pulmonary vascular resistance (PVR) was computed as mean pulmonary arterial pressure (mPAP) subtracted by pulmonary artery wedge pressure and divided by cardiac output.

### 2.3 Cardiac magnetic resonance

#### 2.3.1 Image acquisition

CMR examinations were performed with 1.5T scanners (MAGETOM Aera, Siemens Healthcare, Erlangen, Germany or (Achieva, Philips Healthcare, Best, Netherlands). Standard cine balanced steady-state free precession images of five long-axis views, including RV outflow tract, RV3ch view, LV two-, three-, and four-chamber views, and as well as the SAX covering the whole heart were acquired. Examinations were performed in supine position with a cardiac coil, electrocardiogram-triggered during end-expiratory breath-hold.

Typical image parameters for Siemens were temporal resolution of 46 ms reconstructed to 25 time phases per cardiac cycle, 60° flip angle, 3 ms repetition time, 1.4 ms echo time, and slice thickness 6 mm with 2 mm slice gap; and for Philips, temporal resolution of 47 ms reconstructed to 30 time phases per cardiac cycle, 60° flip angle, 3 ms repetition time, 1.4 ms echo time, and slice thickness 8 mm with no slice gap. In-plane resolutions were typically 1.7–2 × 1.7–2 mm for Siemens and 1.5 × 1.5 mm for Philips.

To acquire late gadolinium enhancement images, gadolinium-based contrast agent (Dotarem, Guerbet, Aulnay-sous-Bois, France and Clariscan, GE Healthcare, Chalfont St. Giles, UK; 0.1–0.2 mmol/kg) was given according to kidney function and clinical practice. Images were acquired 10–20 min after injection in the corresponding standard cine balanced steady-state free precession images in long-axis views and SAX years old at the time of CMRstack. Inversion time was chosen to null remote myocardium [12].

#### 2.3.2 Image analysis

Image analysis and tissue tracking were performed with freely available CMR dedicated software Segment v3.3 (http://segment.heiberg.se) [13]. Image analyses were performed blinded to clinical data by one observer (A.L., 6 years of experience), with a second observer as adjudicator (E.O., >15 years of experience, Society for Cardiovascular Magnetic Resonance level 3).

#### 2.3.3 Global assessment

**Fig. 1:**
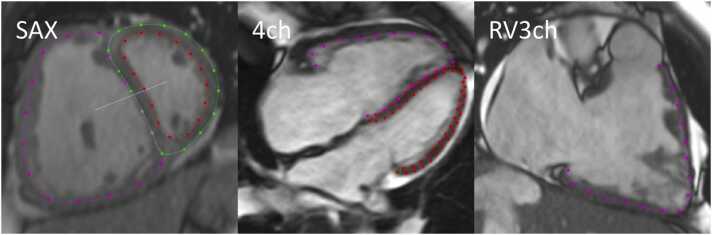
Examples of left ventricular (LV) strain and right ventricular (RV) strain delineations, in end diastole, a patient with pulmonary arterial hypertension. In the left panel, showing a midventricular short-axis slice, the LV endocardium is delineated in red, LV epicardium in green, and RV endocardium in purple. In the middle panel, showing the four-chamber long-axis view, the LV endocardium and epicardium are delineated in red and the RV endocardium in purple. In the right panel, showing the right ventricular three-chamber view, the RV endocardium is delineated in purple. Delineations were performed in end diastole with automatic propagation through the heart cycle. *SAX* short-axis, *4ch* four-chamber, *RV3ch* right ventricular three-chamber

For volumetric assessments, endocardial borders were manually delineated in end diastole and end systole in all SAX slices. End-diastolic volume (EDV), end-systolic volume (ESV), stroke volume (SV), and EF were calculated for both RV and LV. For strain analyses, endocardial contours were manually delineated in end diastole in RV3ch view, 4ch view, and midventricular SAX view at the level of the papillary muscles (Fig. 1). Automated tracking was propagated throughout the heart cycle in each view using feature tracking. If tracking was inadequate, correction in the end-diastolic delineation was performed with a renewed tracking propagation and peak RV global strain computed. RV longitudinal strain in each of the RV3ch and 4ch views, and RV circumferential strain in SAX at midventricular level was assessed as an average of all segments included in the respective views (Graphical abstract).

#### 2.3.4 Dyssynchrony

**Fig. 2:**
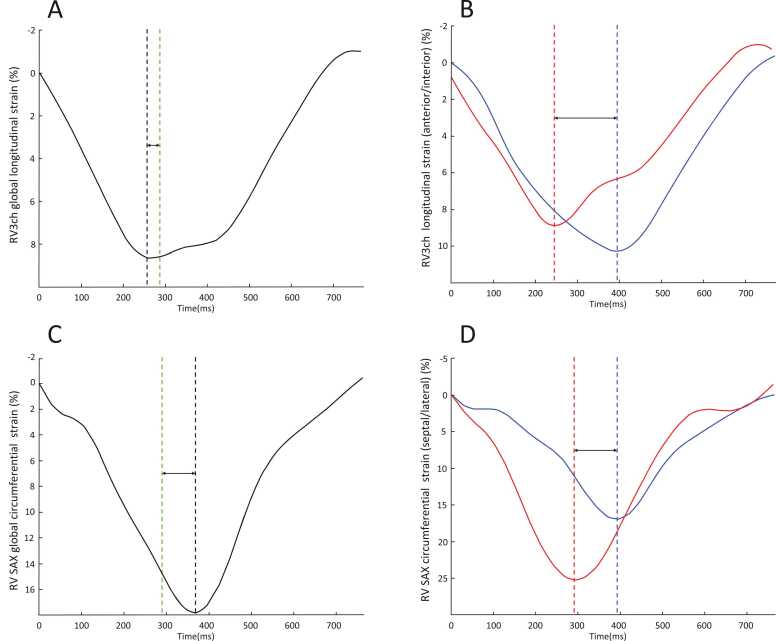
Examples of right ventricular strain plotted on the y-axis and time on the x-axis from two different patients. The left panels (A, C) show global strain. Dyssynchrony was measured as the standard deviation in time between peak systolic strain in the respective walls (red and blue dashed lines). The upper panels show longitudinal strain from RV3ch in a patient without post systolic contraction (A) and with dyssynchrony between anterior and inferior walls (B). The lower panels show circumferential strain from SAX in a patient with post systolic contraction (C) and with dyssynchrony between the septal and lateral walls (D). *SAX* short-axis, *RV3ch* right ventricular three-chamber

The RV was divided into six walls from the three views for dyssynchrony assessment: RV anterior wall and RV inferior wall in RV3ch view, RV lateral wall and septal wall in 4ch view, and RV antero-lateral-inferior wall and septal wall in SAX (Graphical abstract). Time from peak strain and pulmonary valve closure from each wall region was measured, and dyssynchrony was calculated as the standard deviation (SD) indexed to R-wave to R-Wave (RR)-interval (Fig. 2) [8], [9]. If RV3ch view was missing, dyssynchrony from the remaining two views (four walls) was reported. Furthermore, dyssynchrony from two views only (4ch and SAX) and from one view (4ch) or RV3ch was calculated.

#### 2.3.5 Post systolic contraction

PSC was defined as peak strain occurring more than one timeframe after pulmonary valve closure (Fig. 2). Pulmonary valvular closure time was defined from RV outflow tract view (n = 89), and if indefinable from this view, from RV3ch (n = 5) or SAX view (n = 7). PSC presence was noted dichotomously. Duration of PSC was computed as the time difference between peak strain and pulmonary valvular closure time in each of the views, both in ms and in percentage indexed to RR-interval.

#### 2.3.6 Association between dyssynchrony, PSC, and hemodynamics

For association of dyssynchrony and PSC against mPAP and PVR, a subgroup of patients with maximum 30 days between RHC and CMR and no medical change was assessed (n = 56).

### 2.4 Statistical analysis

Statistical analysis was performed with IBM SPSS Statistics for Windows, Version 28 (IBM Corp., Armonk, New York). Normal distribution was assessed using Shapiro-Wilk tests. Continuous variables were expressed as mean ± SD and 95% confidence interval or median and interquartile range [IQR] according to normal distribution. Categorical variables were expressed as absolute numbers and proportion (in percentage). Group comparison was performed with two-sided independent samples T-test or Mann-Whitney U-test. Two-sided chi-square was used for categorical data. Cox regression analysis was used for calculating hazard ratio. Bivariate Cox regression analysis was performed with non-associated variables with p < 0.1. Pearson correlation coefficient was used for computing coefficient of determination (R^2^) for association analyses. A two-sided p-value <0.05 was considered statistically significant.

## 3 Results

One hundred and one patients (58 ± 19 years, 66% (67/101) women) were included for analysis (Table 1). Of 118 patients that fulfilled the inclusion criteria, 17 were excluded due to atrial septal defect (n = 2), atrial fibrillation (n = 6), aortic and mitral valve replacement (n = 1), insufficient image quality (n = 6), or missing more than one of the required views (n = 2).

**Table 1:** Characteristics for patients with PAH, and patients with no death or lung transplantation compared with patients with death or lung transplantation.

	All PAH (n = 101)	No death or LTx (n = 41)	Death or LTx (n = 60)	p-value
Biometrics at CMR				
Age (years)	58 ± 19	47 ± 20	66 ± 15	**<0.01**
Sex; female (%)	66 (67/101)	78 (32/41)	58 (35/60)	**0.04**
BSA (m^2^)	1.8 ± 0.2	1.8 ± 0.2	1.8 ± 0.2	0.5
Heart rate (beats per minute)	78 ± 13	74 ± 12	82 ± 13	**0.02**
NIBP, systolic (mmHg)	128 ± 23^a^	132 ± 22	126 ± 23^a^	0.2
NIBP, diastolic (mmHg)	81 ± 16^a^	83 ± 17	79 ± 14^a^	0.1
NT-proBNP (ng/L)	1978 [3762]^b^	1116 [2178]^c^	2427 [5934]^d^	**0.001**
Right bundle branch block (%)	15 (15/101)	12 (5/41)	17 (10/60)	0.5
Incident/prevalent (%)	72 (73/101)/28 (28/101)	80 (33/41)/20 (8/41)	67 (40/60)/33 (20/60)	0.1
Time between diagnosis to CMR (days)	1 [71]	1 [10]	2 [105]	0.08
Time between CMR and RHC (days)	1 [4]	0 [8]	1 [3]	0.1
RHC^a^				
sPAP (mmHg)	76 ± 21^a^	75 ± 21	76 ± 21^a^	0.7
mPAP (mmHg)	48 ± 14^a^	48 ± 14	48 ± 15^a^	0.9
dPAP (mmHg)	29 ± 11^a^	29 ± 11	29 ± 11^a^	1.0
PAWP (mmHg)	8 ± 4^a^	8 ± 4	8 ± 4^a^	0.8
mRAP (mmHg)	8 ± 6^e^	7 ± 5	8 ± 6^e^	0.4
PVR (wood units)	10 ± 5^a^	10 ± 5	10 ± 5^a^	0.9
Comorbidities at CMR				
Diabetes (%)	23 (23/101)	15 (6/41)	28 (17/60)	0.1
Ischemic heart disease (%)	11 (11/101)	10 (4/41)	12 (7/60)	0.8
Medical treatment at CMR				
PAH treatment (%)	61 (62/101)	49 (20/41)	70 (42/60)	**0.03**
Single therapy (%)	35 (35/101)	29 (12/41)	38 (23/60)	0.3
Dual therapy (%)	27 (27/101)	20 (8/41)	32 (19/60)	0.2
Triple therapy (%)	1 (1/101)	2 (1/41)	0 (0/60)	0.2

Data are presented as mean ± standard deviation, median [interquartile range] or proportion in percent (%)

*LTx* lung transplantation, *PAH* pulmonary arterial hypertension, *BSA* body surface area, *NIBP* non‐invasive systemic blood pressure, *NT-proBNP* N-terminal pro b-type natriuretic peptide, *incident/prevalent*, patients with cardiovascular magnetic resonance imaging (CMR) at time of diagnosis/with known diagnosis, *RHC* right heart catheterization, *sPAP* systolic pulmonary artery pressure, *mPAP* mean pulmonary artery pressure, *dPAP* diastolic pulmonary artery pressure, *PAWP* pulmonary artery wedge pressure, *mRAP* mean right atrial pressure, *PVR* pulmonary vascular resistance, *PAH treatment* treated with PAH-specific medication during CMR (endothelin receptor antagonist, phosphodiesterase type 5 inhibitor or prostanoid), *single therapy* treated with one PAH medication, *dual therapy* treated with two PAH medications, *triple therapy* treated with three PAH medications

p-value: no death or LTx vs death or LTx

Significant p-values are in bold.

a n − 1 (data missing for one subject)

b n = 87

c n = 36

d n = 51

e n − 2 (data missing for two subjects)

Ninety patients were administered gadolinium-based contrast agent during examination. Of these, late gadolinium enhancement was present in 83 patients (92%) (83/90), of which all 83 patients (92%) (83/90) had insertion fibrosis, 6 patients (7%) (6/90) had fibrosis in the septum, and 9 patients (10%) (9/90) in the LV lateral wall. Of the nine patients with lateral fibrosis, two patients (2%) (2/90) had infarction. There was no significant difference in patients without events compared to patients with events with regards to RV insertion fibrosis (p = 0.5), septal fibrosis (p = 0.6), or lateral fibrosis (p = 0.9). Of the analyzed patients, 45 had idiopathic PAH, 7 had heritable PAH, 35 had PAH associated with systemic sclerosis, and 14 had PAH associated with other connective tissue diseases. A majority, 72% (73/101), were incident cases with CMR scans performed within a median of 1 [8] days of diagnosis. Incident cases were defined as newly diagnosed patients and were scanned in conjunction with diagnosis. At time of the CMR examination, 61% (62/101) were on PAH-specific treatment (Table 1). Median follow-up was 37 [51] months.

Sixty patients had an event, of these 55 were deaths and 5 lung transplantations (Table 1). Patients with events were more often male, older, had more PAH-dedicated treatment at CMR, higher heart rate, and N-terminal pro b-type natriuretic peptide than patients without events (Table 1). Sixty-one percent (62/101) of the patients had PAH‐specific medication at CMR, and 94% (94/100) had PAH‐specific treatment after CMR. For one patient we had no data on medication after CMR. Hemodynamic measures obtained during RHC did not differ between patients with or without events (Table 1) Patients with events had lower LV-EDV and LV-ESV compared to patients without events, with no difference in RV-EDV, RV-ESV, or biventricular EF and SV (Table 2). Age was associated with decreased transplantation-free survival in Cox regression analysis (Table 3).

**Table 2:** Cardiac magnetic resonance measures for the whole population and by patients without or with death or lung transplantation.

	All PAH (n = 101)	No death or LTx (n = 41)	Death or LTx (n = 60)	p-value
Left ventricle				
LV-EDV (mL)	125 ± 35	133 ± 39	118 ± 31	**0.03**
LV-ESV (mL)	59 ± 20	64 ± 21	56 ± 20	**0.04**
LV-SV (mL)	65 ± 21	69 ± 23	63 ± 19	0.1
LVEF (%)	53 ± 9	52 ± 8	53 ± 9	0.6
LV-GLS (%)	15 ± 4	15 ± 3	14 ± 4	0.2
LV-GCS (%)	16 ± 4^a^	16 ± 4	17 ± 5^b^	0.2
Right ventricle				
RV-EDV (mL)	229 ± 73	230 ± 69	228 ± 76	0.9
RV-ESV (mL)	154 ± 68	153 ± 63	155 ± 72	0.9
RV SV (mL)	75 ± 21	78 ± 23	73 ± 19	0.2
RV EF (%)	35 ± 12	36 ± 11	34 ± 12	0.6

Data are presented as mean ± standard deviation or median [interquartile range]

*LTx* lung transplantation, *PAH* pulmonary arterial hypertension, *LV* left ventricle, *EDV* end‐diastolic volume, *ESV* end‐systolic volume, *SV* stroke volume, *EF* ejection fraction, *RV* right ventricle, *GLS* global longitudinal strain, *GCS* global circumferential strain

p-value: no death or LTx vs death or LTx

Significant p-values are in bold.

a n = 97

b n = 56

**Table 3:** Univariate and bivariate (adjusted for age) Cox regression analysis for lung transplantation (LTx) or death.

	Univariate HR (95% CI)	Univariate p-value	Bivariate^a^ HR (95% CI)	Bivariate^a^ p-value
Age (per year)	1.04 (1.02–1.05)	**<0.001**		
Dyssynchrony				
Three views (4ch, SAX, and RV3ch) (per %)	1.09 (1.01–1.17)	**0.03**	1.1 (1.01–1.20)	**0.008**
Two views (4ch and SAX) (per %)	1.05 (0.98–1.12)	0.2	-	-
One view (4ch) (per %)	1.00 (0.96–1.05)	1.0	-	-
One view (RV3ch) (per %)^b^	1.02 (0.96–1.09)	0.6		
PSC				
PSC in RV3ch^b^ (y/n)	1.13 (0.56–2.28)	0.7	-	-
PSC in 4ch (y/n)	1.32 (0.79–2.22)	0.3	-	-
PSC in SAX (y/n)	1.48 (0.88–2.50)	0.1	-	-
Time to PSC in RV3ch^b^ (per ms)	1.00 (1.00–1.00)	0.5	-	-
Time to PSC in 4ch (per ms)	1.00 (1.00–1.00)	0.8	-	-
Time to PSC in SAX (per ms)	1.00 (1.00–1.01)	0.2	-	-
Time to PSC in RV3ch indexed to RR-interval^b^ (per %)	1.00 (0.96–1.04)	0.9	-	-
Time to PSC in 4ch indexed to RR-interval (per %)	1.00 (0.98–1.03)	0.6	-	-
Time to PSC in SAX indexed to RR-interval (per %)	1.03 (1.00–1.06)	0.053	1.02 (0.99–1.05)	0.1
RV strain				
RV GLS in RV3ch (per %)	1.03 (0.96–1.12)	0.2	-	-
RV GLS in 4ch (per %)	1.06 (0.99–1.12)	0.09	1.09 (1.02–1.18)	**0.03**
RV GCS in SAX (per %)	1.02 (0.95–1.09)	0.6	-	-

Data are presented as hazard ratio and (95% confidence interval)

Univariate analysis for increased risk of lung transplantation (LTx) or death with continuous variables and bivariate analysis adjusted for age

*HR* hazard ratio for increase in each incremental step, *CI* confidence interval, *PSC* post systolic contraction, *RV3ch* right ventricular three-chamber view, *GLS* global longitudinal strain, *4ch* four-chamber view, SAX, midventricular short-axis slice, *GCS* global circumferential strain from midventricular short-axis slice

Significant p-values are in bold.

a 62 patients with 3 views, 39 patients with 2 views

b n = 62

### 3.1 Dyssynchrony and post systolic contraction

RV dyssynchrony from three views was associated with transplantation-free survival in both univariate and bivariate Cox regression analysis (Table 3). There was no association with survival when dyssynchrony was measured from one or two views (Table 3). There was no difference in RV dyssynchrony between patients with and without events (Table 4).

**Table 4:** Dyssynchrony, post systolic contraction, and RV global strain for the whole population and by patients without or with death or lung transplantation.

	All PAH (n = 101)	No death or LTx (n = 41)	Death or LTx (n = 60)	p-value
Dyssynchrony				
Three views (%)	9 ± 3	8 ± 4	9 ± 3	0.06
Two views (%)	8 ± 4	8 ± 4	9 ± 4	0.3
One view (%)	5 ± 5	5 ± 4	5 ± 5	0.9
PSC				
PSC in RV3ch	30 (48)^a^	13 (43)^b^	17 (53)^c^	0.4
PSC in 4ch	41 (41)	13 (32)	28 (47)	0.1
PSC in SAX	53 (53)	16 (39)	37 (62)	**0.03**
Time to PSC in RV3ch (ms)	61 ± 71^a^	57 ± 71^b^	65 ± 72^c^	0.7
Time to PSC in 4ch (ms)	52 ± 74	49 ± 79	55 ± 72	0.7
Time to PSC in SAX (ms)	61 ± 72	47 ± 69	71 ± 72	0.1
Time to PSC in RV3ch indexed to RR-interval (%)	8 ± 9^a^	7 ± 9^b^	8 ± 9^c^	0.6
Time to PSC in 4ch indexed to RR-interval (%)	7 ± 10	6 ± 11	8 ± 10	0.5
Time to PSC in SAX indexed to RR-interval (%)	8 ± 9	6 ± 8	9 ± 9	
RV strain				
RV GLS in RV3ch (%)	14.2 ± 6^a^	15.1 ± 5^b^	13.3 ± 6^c^	0.2
RV GLS in 4ch (%)	14.8 ± 4	15.7 ± 4	14.2 ± 4	0.09
RV GCS in SAX (%)	8.7 ± 4	8.4 ± 4	9.0 ± 4	0.4

Data are presented as mean ± standard deviation or absolute numbers and proportion in percentage (%) Dyssynchrony (SD of the different walls/RR-interval in %) from one, two, and three views, post systolic contraction (PSC) and global strain in right ventricular (RV) three-chamber view (RV3ch), four-chamber view (4ch), and midventricular short-axis slice (SAX)

*LTx* lung transplantation, *RV* right ventricle, *GCS* midventricular global circumferential strain, *SAX* midventricular short-axis slice. *PSC* post systolic contraction, *GLS* global longitudinal strain, *4ch* four-chamber view, *3ch* three-chamber view, *PAH* pulmonary arterial hypertension

p-value: no death or LTx vs death or LTx

Significant p-values are in bold.

a n = 62

b n = 30

c n = 32

Dyssynchrony from three views was significantly higher in patients with insertion fibrosis (p = 0.03), but not with septal fibrosis (p = 0.4) or LV lateral wall fibrosis (p = 0.09), compared to patients without fibrosis.

Dyssynchrony from three views correlated weakly with RV EF (R^2^ = 0.17, p < 0.001), RV SV (R^2^ = 0.04, p = 0.04), RV GLS from RV3ch (R^2^ = 0.12, p = 0.01), and RV GLS from 4ch (R^2^ = 0.21, p < 0.001).

PSC was present in 48% (30/62) of patients in RV3ch, 53% (53/101) in SAX view, and 41% (41/101) in 4ch view (Table 4). PSC was more prevalent SAX view in patients with events than without, with no difference in RV3ch or 4ch (Table 4). In bivariate regression analysis, there was no association between survival and presence of PSC or duration of PSC in any views (Table 3).

Patients with and without events did not differ regarding LV (Table 2) or RV strain in any views (Table 4). RV GLS from 4ch was associated with transplantation-free survival for the whole population (Table 3), while RV global circumferential strain from SAX or GLS from RV3ch showed no association with survival in Cox regression analysis.

Dyssynchrony in three views was determined by mPAP (9%) and PVR (23%) (Table 5). Dyssynchrony in one and two views and time to PSC was determined by PVR in 13%–16%, but not by mPAP. RV GLS in RV3ch and 4ch views was also determined by mPAP (20%) and PVR (43%–48%).

**Table 5:** Correlations between dyssynchrony, PSC, and strain with mPAP and PVR.

	mPAP		PVR	
	R^2^	p-value	R^2^	p-value
Dyssynchrony				
Three views^a^	0.09	**0.03**	0.225	**<0.001**
Two views	0.02	0.3	0.13	**0.008**
One view	0.04	0.2	0.16	**0.002**
PSC				
Time to PSC in RV3ch^b^	0.00	0.9	0.10	0.055
Time to PSC in 4ch	0.003	0.7	0.009	0.5
Time to PSC in SAX	0.005	0.6	0.06	0.1
Time to PSC in RV3ch indexed to RR-interval^b^	0.001	0.9	0.14	**0.03**
Time to PSC in 4ch indexed to RR-interval	0.005	0.6	0.013	0.4
Time to PSC in SAX indexed to RR-interval	0.008	0.5	0.058	0.08
RV strain				
RV GLS in RV3ch^b^	0.2	**0.008**	0.43	**<0.001**
RV GLS in 4ch	0.21	**<0.001**	0.48	**<0.001**
RV GCS in SAX	0.064	0.09	0.11	**0.01**

Data are presented as coefficient of determination (R^2^).

Correlations between dyssynchrony from one, two, and three views, PSC and strain in right ventricular (RV) three-chamber view (RV3ch), four-chamber view (4ch), and midventricular short-axis slice (SAX) with mPAP and PVR

*mPAP* mean pulmonary arterial pressure, *PVR* pulmonary vascular resistance, *R^2^* coefficient of determination, *PSC* post systolic contraction, *RV3ch* right ventricular three-chamber view, *GLS* global longitudinal strain, *4ch* four-chamber view, *SAX* midventricular short-axis slice, *GCS* global circumferential strain from midventricular short-axis slice

Significant p-values are in bold.

a 33 patients with 3 views, 23 patients with 2 views

b n = 33

## 4 Discussion

This study demonstrated that RV dyssynchrony from multiple views by CMR was a prognostic marker for transplantation-free survival in PAH. This could potentially have future implications for risk stratification in PAH and treatment decisions. PSC did, however, not improve prognostication.

### 4.1 Dyssynchrony

Dyssynchrony from the six RV walls in the three views (RV3ch, 4ch, and SAX) was associated with decreased transplantation-free survival in both univariate and bivariate analysis. An increase of dyssynchrony by 1% was associated with a 10% increased risk of lung transplantation or death. To our knowledge, this is the first study showing an association between RV dyssynchrony and survival. Previously, in an echocardiographic study of PAH patients, RV dyssynchrony was correlated with World Health Organization (WHO) functional class and 6-minute walked distance as well as RV area, fractional area change and hemodynamic measurements but not directly to outcome [6].

There are some possible explanations as to why RV dyssynchrony from multiple views was associated with transplantation-free survival. RV dilation predominantly occurs in the anteroinferior direction in PAH [14] and RV3ch includes the anterior and inferior walls. The anteroinferior dilation is emphasized by an augmentation of RV sphericity, which in turn is a predictor of clinical deterioration in PAH [14]. In the 4ch view, only septal and lateral RV walls are assessed, and thus, essential parts of dilation and wall motion abnormalities could be missed [7], [8], [9]. RV dyssynchrony including RV3ch might be more sensitive to RV functional deterioration as the septum is not included and therefore is less susceptible to LV interaction. However, dyssynchrony from RV3ch alone was not associated with decreased survival. This could be partially impacted by the reduced percentage of available RV3Ch. Nevertheless, incorporating the anterior and inferior walls by adding RV3ch to conventional views (4ch and SAX) could still provide a more comprehensive RV examination of PAH patients.

### 4.2 Post systolic contraction

PSC was present in about half of the patients, however, neither PSC presence nor duration was associated with transplantation-free survival in the present study. On the other hand, PSC was associated with PVR. That PSC is a sign of hemodynamic status in keeping with the duration of the RV post systolic isovolumic period has been associated with PVR and systolic pulmonary arterial pressure [7]. PSC exhibits different curve patterns. These patterns have been shown associated with hemodynamic conditions, clinical worsening, and death, of which clinical worsening was a substantial part of the combined outcome [15]. Altogether, PSC could be a potential indicator for clinical worsening and hemodynamic status rather than a prognostic marker.

### 4.3 Strain

Peak global circumferential, longitudinal, and radial strain are shown lower in PAH compared to healthy controls [2], [3], [4], [5]. In the present study, survival was associated with 4ch RV GLS. This is in agreement with previous studies in PAH where RV GLS by echocardiography was associated with survival [16], [17] and by CMR was associated with a combined endpoint of death, lung transplantation, and worsening WHO functional class [4]. In a multi-modal context, it should be noted that RV GLS measured by echocardiography or CMR is not fully interchangeable in PAH [18]. Neither is it recommended to use strain values interchangeably among vendors due to intervendor variability [19], [20].

### 4.4 Relation to hemodynamic measures

We found that dyssynchrony in three views correlated with mPAP and PVR, but not with one and two views or time to PSC. There was a stronger association with PVR than mPAP. Since PVR infers disease severity, the stronger association to PVR rather than mPAP could be expected [1], [21]. However, the associations were weak, and thus the clinical implications are limited.

## 5 Limitations

Some limitations should be noted. First, this is a retrospective study with risk for bias in the selection of study participants. As clinical routine in our tertiary center is to refer all patients for CMR, the likelihood for selection bias is mitigated. However, the most severely ill patients may not have been referred for, or not able to complete, a CMR. Second, RV3ch view was included in the clinical protocols 2012, why only 62 of 101 patients had all three views. Despite this limitation, including RV3ch view to the dyssynchrony assessment still showed significance, which strengthens the notion of comprehensive assessment from multiple views and improves the prognostication. Third, we changed the scanner in 2015, why two different vendors were used in this study. Theoretically, different scanners might generate different strain results even when using the same software, yet data regarding inter-scanner reproducibility from CMR is lacking. Fourth, temporal resolution could influence both strain and PSC analyses, and a disadvantage with CMR strain compared to echocardiography is the lower temporal and spatial resolution as well as challenges of strain assessment on a segmental level [22]. On the other side, the ability to visualize RV comprehensively without restrictions in the acoustic windows is an advantage with CMR [23].

Due to the high number of variables included in the study, we did not perform multivariate analysis with all included variables but only bivariate analysis with age. It would be of worth to determine if dyssynchrony or PCS could provide added prognostic value to RV strain parameters in future prospective multicenter studies.

## 6 Conclusions

Dyssynchrony from three views was associated with outcome in PAH whereas dyssynchrony from one or two views and PSC were not. PSC has potential as an indicator of clinical worsening and hemodynamic status, rather than a prognostic marker. Our study implies that comprehensive dyssynchrony assessment from multiple views, including RV3ch, may help improve prognostication in PAH.

## Funding

This study was funded by research grants from Swedish Research Council, Stockholm, Sweden [grant number 2021-02779]; Region Skåne (ALF), Lund, Sweden; Swedish Society of Medicine, Stockholm, Sweden [grant number SLS-961558]; Swedish Heart and Lung Foundation, Stockholm, Sweden [grant number 20190576, 20210337, 20220449, 20230550]; 10.13039/501100003173Crafoord Foundation, Lund, Sweden [grant number 200681, 20230611]; 10.13039/501100003252Lund University, Lund, Sweden; and Skåne University Hospital Foundations.

## Author contributions

**Göran Rådegran:** Writing—review and editing, Supervision, Data curation. **Håkan Arheden:** Writing—review and editing, Supervision. **Ellen Ostenfeld:** Writing—review and editing, Supervision, Resources, Project administration, Methodology, Conceptualization. **Anthony Lindholm:** Writing—review and editing, Writing—original draft, Project administration, Methodology, Investigation, Formal analysis, Data curation, Conceptualization. **Barbro Kjellström:** Writing—review and editing, Supervision, Project administration.
